# No Detectable Effect on Visual Responses Using Functional MRI in a Rodent Model of α-Synuclein Expression

**DOI:** 10.1523/ENEURO.0516-20.2021

**Published:** 2021-05-14

**Authors:** Freja Gam Østergaard, Christian Stald Skoven, Alex R. Wade, Hartwig R. Siebner, Bettina Laursen, Kenneth Vielsted Christensen, Tim B. Dyrby

**Affiliations:** 1 Circuit Biology, H. Lundbeck A/S, Valby DK-2500, Denmark; 2 Department of Psychology, The University of York, Heslington, York YO10 5DD, United Kingdom; 3 Danish Research Centre for Magnetic Resonance, Centre for Functional and Diagnostic Imaging and Research, Copenhagen University Hospital - Amager and Hvidovre, Copenhagen 2650, Denmark; 4 Department of Applied Mathematics and Computer Science, Technical University of Denmark, Kongens Lyngby 2800, Denmark

**Keywords:** α-synuclein, fMRI, rat, superior colliculus, vision

## Abstract

Parkinson’s disease (PD) is a progressive neurodegenerative disease that is typically diagnosed late in its progression. There is a need for biomarkers suitable for monitoring the disease progression at earlier stages to guide the development of novel neuroprotective therapies. One potential biomarker, α-synuclein, has been found in both the familial cases of PD, as well as the sporadic cases and is considered a key feature of PD. α-synuclein is naturally present in the retina, and it has been suggested that early symptoms of the visual system may be used as a biomarker for PD. Here, we use a viral vector to induce a unilateral expression of human wild-type α-synuclein in rats as a mechanistic model of protein aggregation in PD. We employed functional magnetic resonance imaging (fMRI) to investigate whether adeno-associated virus (AAV) mediated expression of human wild-type α-synuclein alter functional activity in the visual system. A total of 16 rats were injected with either AAV-α-synuclein (*n* = 7) or AAV-null (*n* = 9) in the substantia nigra pars compacta (SNc) of the left hemisphere. The expression of α-synuclein was validated by a motor assay and postmortem immunohistochemistry. Five months after the introduction of the AAV-vector, fMRI showed robust blood oxygen level-dependent (BOLD) responses to light stimulation in the visual systems of both control and AAV-α-synuclein animals. However, our results demonstrate that the expression of AAV-α-synuclein does not affect functional activation of the visual system. This negative finding suggests that fMRI-based read-outs of visual responses may not be a sensitive biomarker for PD.

## Significance Statement

We injected an adeno-associated virus (AAV) vector in rats to induce unilateral expression of human wild-type α-synuclein in the substantia nigra, and in the ipsilateral striatum and superior colliculus (SC). This did not affect functional activation of SC as probed with functional magnetic resonance imaging (fMRI). This negative finding discourages the use of functional brain mapping of visually evoked activity as an indicator of regional expression of human α-synuclein.

## Introduction

Parkinson’s disease (PD) is the second most common neurodegenerative disorder (Wirdefeldt et al., 2011) affecting millions of people worldwide (Thomas and Beal, 2007). Although the disease is primarily considered to be sporadic, there are familial versions caused by a mutation in a single gene (Klein and Westenberger, 2012). These hereditable forms of PD have led to the discovery of several susceptibility genes, such as the *SNCA* gene (park1/4) coding for α-synuclein. Aggregation of α-synuclein into fibrils and Lewy bodies is hypothesized to develop long before the diagnostic symptoms of tremor and postural instability (Noyce et al., 2016). These aggregates have been proposed to give rise to symptoms early in the course of the disease, consequently, they may potentially serve as biomarkers for the progression.

This study focuses on the visual system as several studies have shown that the visual system is affected in PD patients (Davidsdottir et al., 2005; Ekker et al., 2017; Satue et al., 2017). Moreover, these changes have so far failed to provide an unambiguous estimate of disease severity (Ridder et al., 2017). This is likely because of PD being diagnosed at a very late stage of disease progression, where patients are prone to suffer from a wide range of visual changes related to normal aging (Ekker et al., 2017). However, evidence of PD-related changes in vision was provided by postmortem studies showing α-synuclein aggregation in the retinae of PD patients (Bodis-Wollner et al., 2014). This finding lead to the hypothesis that α-synuclein aggregation may cause changes in visual responses measured in patients with PD.

A rodent model of α-synuclein expression in the substantia nigra pars compacta (SNc), mediated by the adeno-associated virus (AAV) has shown to decrease the number of spines of the dopaminergic cells, which in turn have shown to influence the neuronal firing pattern in the striatum (Andersen et al., 2018). In addition to the striatum, the SNc also projects to the superior colliculus (SC) which is the primary region for visual processing in rats (Sefton et al., 2014). Further it was shown by Østergaard and colleagues that this model also exhibits changes in the latency of the visual evoked potential (VEP) measured in the SC (Østergaard et al., 2020). The neuronal firing pattern was measured invasively using electrophysiology making it infeasible to apply in humans. Hence, a non-invasive technique is desirable. MRI is a non-invasive bioimaging modality, which have been used to investigate regional changes in brain structure of PD patients (Lehéricy et al., 2014). Most MRI-based studies of PD patients have focused on changes in brain structure (Niethammer et al., 2012; Weil et al., 2016). Functional MRI (fMRI) measures the local changes in blood oxygenation related to neuronal activity and has been applied in PD patients to study changes in neuronal function. One of these studies have shown changes in the iron load of cortex and SNc in PD patients (Pyatigorskaya et al., 2014). fMRI may also detect neuronal changes in the dopamine release after a pharmacological challenge in the rodent PD model (Kuebler et al., 2017) also used by Andersen and colleagues (Andersen et al., 2018). This model mimics the mechanistic changes of expressing human wild-type α-synuclein in neurons of the substantia nigra (Decressac et al., 2012).

The aim of this study was to use blood oxygen level-dependent (BOLD) fMRI with a visual flickering full-field illumination stimulation to examine potential functional consequences of expressing human wild-type α-synuclein in the rodent. AAV carrying DNA encoding human wild-type α-synuclein (*hSNCA*) was unilaterally injected in SNc in rats with the aim of exogenously expressing the human α-synuclein protein and was thereby expected to cause an asymmetry of the BOLD response. In 2013, Bailey et al. (2013) described how the cortex responds differently to frequencies above and below a presentation rate of 10 Hz, therefore we have chosen to use presentation rates of 1 and 14 Hz. The response to one frequency could be more sensitive to changes introduced by expressing human α-synuclein compared with the response to the other frequency, as responses to these two frequencies may be governed by different mechanisms (Bailey et al., 2013). We use the regions of interest (ROIs) of the rodent visual system and then compared them to the olfactory bulb (OB). The OB is used as a control region, as this region is easy to define and neutral with regards to visual stimuli.

## Materials and Methods

### Subjects

All animal experiments were conducted in accordance with the European Communities Council Directive (86/609/EEC), and in accordance with Danish law on care of laboratory animals. The protocols were approved by the Danish Animal Experiments Inspectorate (Forsøgsdyrstilsynet) before the initiation of the study.

A total of 20 female Sprague Dawley (SD) rats (Taconic) weighing ∼225 g (corresponding to 10 weeks of age) on arrival were included in the study. The rats underwent surgery and cylinder test at one test site and were transported to another for MRI scanning. The animals acclimatized to the new housing facility for at least two weeks before imaging. The homecage environment including light cycle, cages and enrichment was similar at the two housing facilities. Both had a 12/12 h light/dark cycle (lights on at 6 A.M.). The cages were enriched with nesting material and a red plastic shelter. Access to food (chow) and water was *ad libitum*.

### Surgery

Before surgery, each rat was anesthetized using subcutaneous injections of Hypnorm (Lundbeck), midazolam 5 mg/ml (B. Braun), and saline in a 2:1:1 ratio yielding an injection volume of 1.7 ml/kg. The rat was placed in a stereotaxic frame and 0.1 ml Marcain (2.5 mg/ml bupivacaine, AstraZeneca) was administered locally and subcutaneously before incision. A small craniotomy (Ø = 1 mm) was made over the SNc of the left hemisphere (AP: −5.5 mm; ML: +2.0 mm; DV: −7.2 mm relative to bregma; Paxinos and Watson, 1998) to allow for injection of 3 μl AAV 2/5 (3 × 10^10^ GC/ml; Vector biolabs), carrying human wild-type α-synuclein (*hSNCA*) using methodology as described previously (Andersen et al., 2018). Half of the animals acted as controls by having the empty viral vector injected. The animals were postoperatively treated with buprenorphine 0.05 mg/kg every eighth hour for 4 d. The rats were single-housed after the surgery.

### Cylinder test

To validate the expression of human wild-type α-synuclein in the basal ganglia, the rats were tested for motor asymmetry 10 weeks after surgery. The animals were recorded with a video camera for 5 min, while they were freely moving in a Plexiglas cylinder. Paw touches on the side of the cylinder were counted offline and blinded to the group. The ratio of touches of contralateral paw to total touches was computed. Animals with an expression of human α-synuclein were hypothesized to use the contralateral paw less than the control animals.

### MRI

#### Preparation for fMRI

Five months after the viral injection, the rats were anesthetized and scanned in a 7T Bruker Biospec (Bruker BioSpec 70/20 USR) MRI scanner with an 80-mm radio frequency (RF) transmit quadrature coil and a 20 mm surface receive coil. The surface receive coil was fixed on top of the head using masking tape. The position of the surface ensured good signal coverage of the whole brain having the largest signal sensitivity in the ROIs: the SC and visual cortex. Sticky tack (Bantex) was placed in the ears of the rats to reduce harmful effects of MR scanner noise during scanning. The animals were scanned at the same time of day, on separate days, to minimize variation caused by the circadian rhythm.

##### Anesthesia during scan session

To reduce the unwanted effect of isoflurane on the BOLD signal, we used a combination of low dose isoflurane and dexmedetomidine (Dexdormitor, Orion Pharma). Anesthesia was induced with isoflurane at 5% and adjusted to 2.5% while placing a tail-vein catheter. Subsequently, dexmedetomidine was infused at 0.05 mg/kg/h (Pawela et al., 2009). When the heart rate decreased to ∼200 bpm, isoflurane was adjusted to 0.5% (in 1 l of oxygen:medical air, 8:2). The animals breathed spontaneously, and the respiration rate of the animals was continuously monitored by a respiration sensor pad (SA Instruments). Blood oxygen saturation and heart rate was monitored transcutaneously using pulse oximetry (Nonin). The core temperature of the animals was monitored with a rectal probe and used as feedback to maintain a constant temperature of 37°C using heated air (SA Instruments). After 1 h of infusion, the infusion rate of dexmedetomidine was increased to 0.1 mg/kg/h. This concentration is lower than what most literature recommend (Pawela et al., 2009) because the vasoconstrictive effects of dexmedetomidine made measuring SpO2 challenging, and this lower concentration turned out to be sufficient.

#### MRI protocols

Since the structural T1 weighted is not affected by anesthesia it was acquired before the fMRI. Before acquiring the actual fMRI, online fMRI correlation analysis was applied at regular time intervals using a visual stimulus paradigm. Online fMRI correlation analysis used the Analysis of Functional NeuroImages (AFNI) software integrated with the fMRI sequence (Cox, 1996). The online fMRI analysis was used to determine when the impact of isoflurane on the neuronal response and BOLD signal was diminished, i.e., washed out. Typically, robust regional BOLD responses were obtained after 180 min.

Structural MRI to be aligned with fMRI included a whole brain 3D T1-weighted fast low angle shot (FLASH) MRI [repetition time (TR) = 1500 ms, echo time (TE) = 8 ms, inversion time (IR) = 103,338.0 ms, isotropic 0.2-mm^3^ voxels field of view of 175 × 80 × 175 mm^3^, matrix size = 35 × 16 × 35).

The echoplanar image (EPI) MRI had an in-plane voxel size of 0.313 × 0.313 mm with slice thickness of 0.500 mm, TR = 1500 ms, and TE = 8.35 ms, slice gap = 0. The Ernst flip angle was determined to 64°, based on T1 relaxation measurement of cortical gray matter obtained from a separate session (data not shown). 42 coronal slices covered the range from the caudal part of the OB to the caudal part of the cerebellum.

#### Visual stimulation paradigm for fMRI

Visual stimulation was provided by five optical fibres (diameter of 1.5 mm) of “warm white” light provided by a light emitting diode (LED) source placed outside the scanner. The optical fibres were placed in front of the animal head as an array. The light intensity during the stimulation was kept at 20 lx, measured at the level of the eyes using a LED luxmeter (Extech).

The stimulation paradigm used a block design that was generated by a micro1401 data acquisition unit (Cambridge Electronic Design Ltd.) controlled from Spike2 version 7.20 synchronized to TTL triggers from the scanner. The block design included six task blocks per trial repeated five times, where each task block lasted for 21 s, equivalent to 14 TRs. Each task block consisted of either 1- or 14-Hz light flicker. These two frequencies were chosen as one should be just above 10 Hz, while the other should be well below, as 10 Hz is considered to be the threshold for saturation of the visual cortex (Van Camp et al., 2006). Between task blocks there were a pause of 14 TRs (21 s). The visual stimulation paradigm was repeated five times at each stimulation frequency yielding a total scan time of ∼4 h.

#### fMRI preprocessing

MRI data were analyzed using the FMRIB software library (FSL; Woolrich et al., 2009). To make a standard brain template, the EPIs were aligned within subject using FMRIB’s linear image registration tool (FLIRT) and then averaged across animals to construct a standard brain template. This template was used for registration of the data for later analysis.

Visual inspection caused exclusion of four rats, as these could not be aligned to the standard template leaving 16 to be analyzed; nine controls and seven AAV-α-synuclein animals.

### Statistical analysis

fMRI data were analyzed using a three-level statistical analysis pipeline implemented in the FSL expert analysis tool (FEAT). In the first level, the time course of each voxel was fitted with a general linear model (GLM) to produce a statistical map of the *z* scores of the correlation for each voxel within each of the scans. Each voxel was corrected for family-wise error with a p-threshold of *p* < 0.05. Spatial smoothing was then applied using a Gaussian kernel with full width half maximum of 0.5 mm. In the second level, the z-statistical maps were averaged for each stimulation frequency within each subject yielding two averaged z-statistical maps per animal. Each voxel within the averaged statistical map was corrected for family-wise error with a *p* threshold of *p* = 0.05. At the third and final level, the averaged statistical maps were averaged within the two groups for each of the two stimulation frequencies. The group statistical maps were compared using an unpaired comparison of the z-statistical maps between the two groups within each frequency paradigm. For visualization, the statistical maps were aligned to the standard brain template (described above) and then superimposed on the high-resolution structural MRI scan.

Structural ROIs of the SC and visual cortex were defined manually based on rat brain atlases (Papp et al., 2014; Kjonigsen et al., 2015; Sergejeva et al., 2015). The OB was used as a control ROI. For each group there were ROIs for OB, right SC, left SC, right VC and left VC. The percent change of the BOLD within the ROIs were extracted from the z-statistical map of each rat using FEAT query. Further, estimation statistics were conducted using the website https://www.estimationstats.com/#/, which is based on the methods described in (Ho et al., 2019). Here, the data were compared with the common control method within each stimulus paradigm, tested using a two-sided permutation *t* test and corrected for multiple comparisons. The resulting effect sizes and confidence intervals (CIs) are listed in Table 1.

**Table 1.** Outcome of estimation statistics of the BOLD responses from each ROI

Figure	Stimulus	Control ROI	ROI	Effect size	CI (width 95.0%)		*p* value
					Lower bound	Upper bound	
3	1 Hz	OB control	OB α-synuclein	−0.000905	−0.00444	0.0502	0.981
			VC control contra-	0.493	0.344	0.651	0.00022
			VC control ipsi-	0.415	0.255	0.568	0.00099
			VC α-synuclein contra-	0.336	−0.0667	0.609	0.0452
			VC α-synuclein ipsi-	0.429	0.127	0.68	0.0044
			SC control contra-	1.2	0.885	1.52	0.000123
			SC control ipsi-	1.15	0.816	1.57	0.000498
			SC α-synuclein contra-	1.28	0.917	1.5	0.000133
			SC α-synuclein ipsi-	1.22	0.958	1.4	0.000036
4	14 Hz	OB control	OB α-synuclein	−0.038	−0.243	0.14	0.722
			VC control contra-	−0.424	−0.785	−0.0208	0.0496
			VC control ipsi-	−0.257	−0.528	0.0135	0.0926
			VC α-synuclein contra-	−0.258	−0.638	0.0227	0.139
			VC α-synuclein ipsi-	−0.414	−0.714	−0.182	0.0012
			SC control contra-	1.29	0.744	1.8	0.0006
			SC control ipsi-	1.22	0.66	1.9	0.0006
			SC α-synuclein contra-	1.38	0.585	2.01	0.001
			SC α-synuclein ipsi-	1.21	0.273	1.72	0.0026

The 95 % CI are based on a two-sided permutation t-test with 5000 bootstraps. ROI; region of interest, Contra-; contralateral to the injection, Ipsi-; ipsilateral to the injection, OB; olfactory bulb, VC; visual cortex, SC; superior colliculus.

### Immunohistochemistry

At the end of the scan session, the animal was given a bolus 3 ml/kg of Hypnorm and midazolam was administered SC. The animals were perfusion fixated with intracardial potassium PBS (KPBS) for 3 min, followed by 4% paraformaldehyde (PFA) premade with methanol. After 10–15 min, the rat was decapitated, and the brain extracted. The brain was immersed in 4% PFA for 2 h, then in phosphate buffer (PBS) with <1% PFA for 24 h, and finally in KPBS.

Brains were fixed in 30% sucrose for 72 h before slicing. Once fixed, they were dried and frozen with dry ice and placed in a freezing microtome (Leica CM3050S) for 30 min; 40-μm-thick coronal slices were cut and stored in KPBS. All slices were stained within 5 d.

Brain sections were stained to validate the expression pattern of the human wild-type SNCA protein. Before staining, the tissue was quenched with hydrogen peroxide 3% to remove any traces of blood. The slices were washed and incubated overnight with 4B12 (Thermo Scientific) for human α-synuclein as the primary antibody (concentration 1:1000). Subsequently, the slices were washed and incubated with biotin conjugated anti-mouse secondary antibody (Jackson ImmunoResearch) and exposed using a 3,3´-diaminobenzidine (DAB) reaction for ∼20 min. The stained slices were placed on gelatin covered glass slides, and visually examined using a light microscope (Axio scope.A1, Carl Zeiss Microimaging).

## Results

### Motor assessment of α-synuclein expression

**Figure 1.**
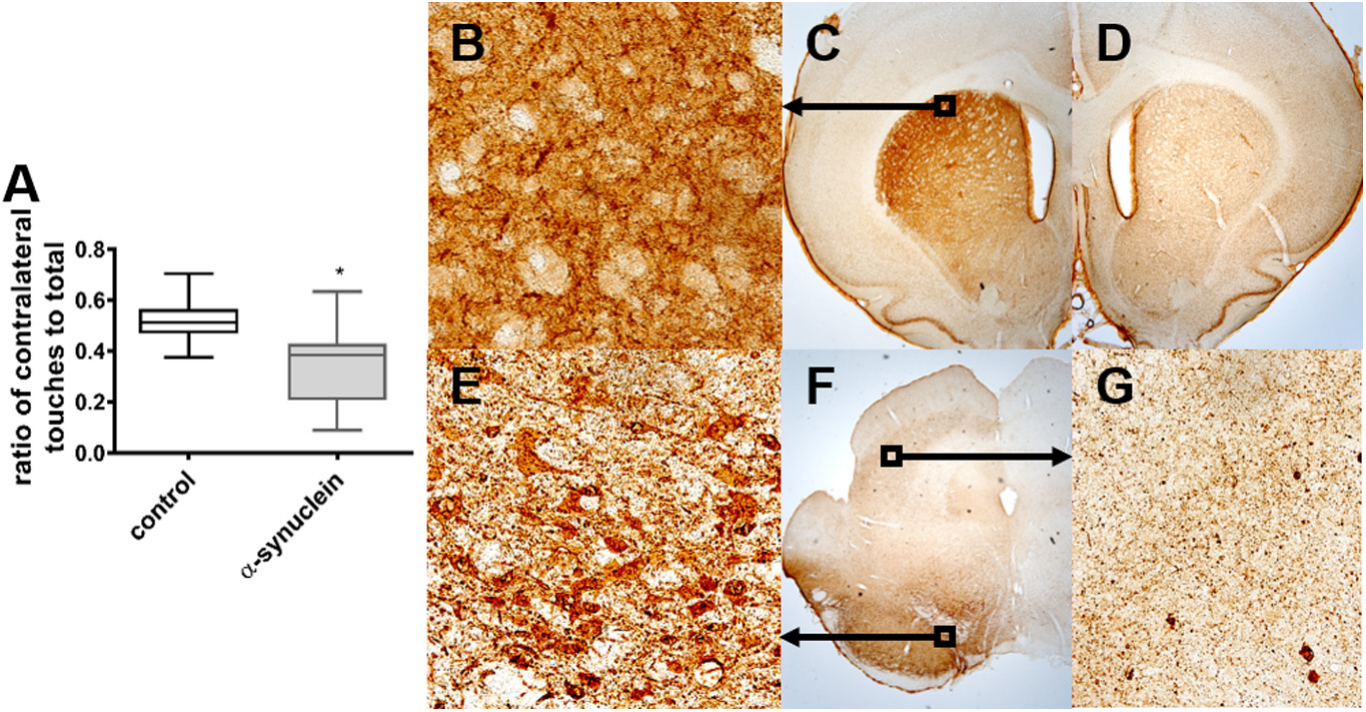
Validation of the AAV-α-synuclein model. **A)** Cylinder test of the animals showing the ratio of touches by the contralateral paw to the total number of touches. The control animals (in black) have a ratio of 0.51 indicating equal use of both paws. The α-synuclein animals (in grey), have a ratio of 0.35 indicating that they use the paw contralateral of the injection less than the ipsilateral paw. **(B-G)** Representative coronal sections stained for α-synuclein. **B)** 40x magnification of striatal neurons showing α-synuclein-positive inclusions. **C)** 2,5x magnification of striatum showing α-synuclein immunoreactivity. **D)** 2,5x magnification of striatum contralateral to the injection of AAV-α-synuclein. **E)** 40x magnification showing α-synuclein in cell somas in the substantia nigra. **F)** 2,5x magnification of section showing the substantia nigra and the superior colliculus. **G)** 40x magnification showing α-synuclein immunoreactivity in the SC. The slices were stained with 4B12 (human WT alpha-synuclein).

To validate the expression and impact of human α-synuclein in the rat basal ganglia, α-synuclein and control animals were tested for motor asymmetry in the cylinder test (Andersen et al., 2018). Figure 1*A* shows the change in the use of the forepaw contralateral to the injection site. Control animals used both paws equally (ratio of 0.5), while α-synuclein animals had a significantly different mean ratio of affected/total touches ratio of 0.35 (*t*_(14)_ = 2.8, *p* = 0.01). The cylinder test confirmed that the function of the striatum was unilaterally affected in the animals that received the AAV-*hSNCA* injection, as expected with successful injection of viral particles carrying the *hSNCA*-expressing vector.

### Validation of expression of human α-synuclein

Immunohistochemical evaluation conducted after fMRI, showed immunoreactivity toward human α-synuclein in the SNc (Fig. 1*E*,*F*), in areas surrounding the SNc, and in the striatum ipsilateral (Fig. 1*B*,*C*) to the injection site. Interestingly, small inclusions of immunoreactivity were observed in the optic layer of the ipsilateral SC (Fig. 1*G*). Minor immunoreactivity was observed along the edges of the tissue (Fig. 1*C*,*D*) which were not specific to the injected hemisphere thus considered as unspecific immunoreactivity. Confirmation of α-synuclein in areas ipsilateral to the injected hemisphere supports the observations from the cylinder test.

### fMRI of the visual system

**Figure 2.**
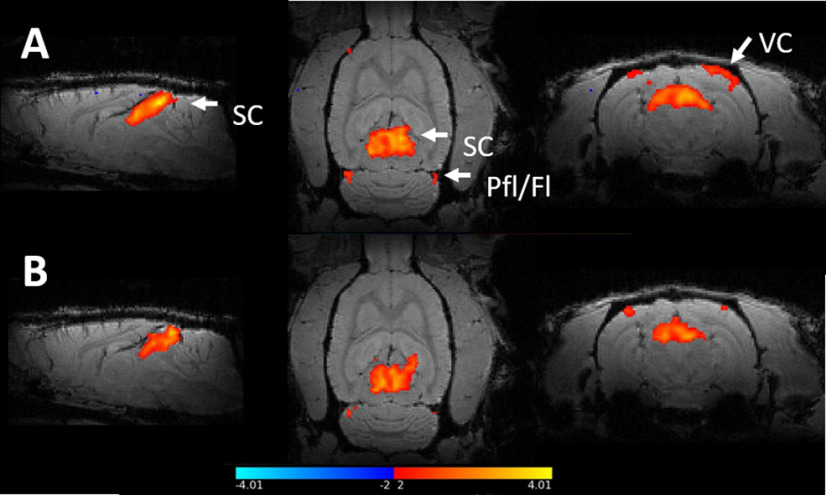
Statistical group maps of BOLD fMRI responses to visual stimulation. The colour bar shows the thresholds for the z-scores, as anything between -2 and 2 is considered insignificant. The figure shows the group averages of activation as computed in the level 3 FEAT analysis and registered to a high-resolution anatomical scan. **A)** control group **B)** α-synuclein group. Visual stimulation with flickering light robustly activated the superior colliculus (SC) and in the visual cortex (VC). Additionally, the paraflocculus/flocculus complex (Pfl/Fl) was activated.

The z-statistical maps of the two groups are shown in Figure 2 registered to a high-resolution anatomic scan. Overall, there were detectable responses in both the SC and the VC during light stimulation in both groups of rats. A modest activation of cerebellar flocculus complex was also observed. This is in line with previous studies where visual stimulation has been applied (Van Camp et al., 2006). Additionally, there was a response in the lateral geniculate nucleus (LGN), as expected (Bailey et al., 2013).

**Figure 3.**
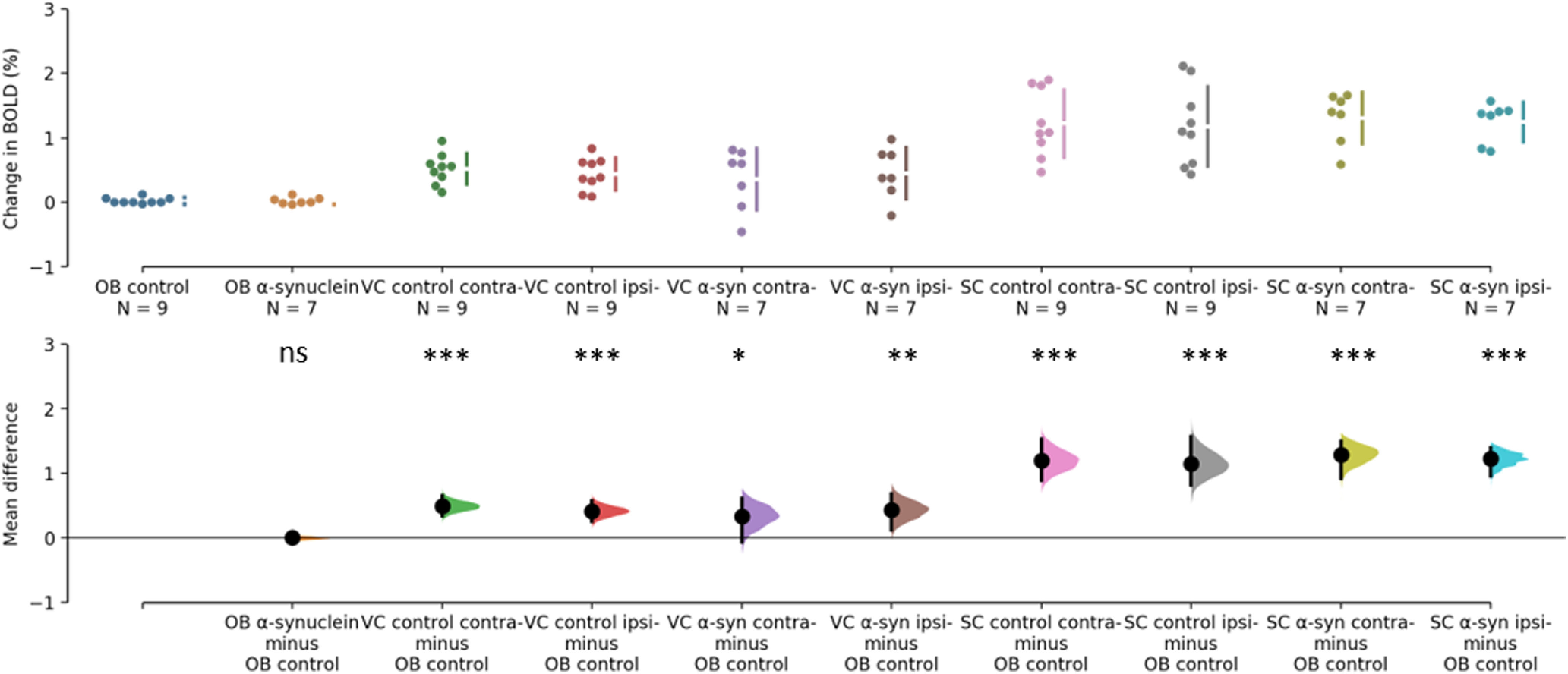
Estimation statistics of percent change in BOLD during 1 Hz stimulation. The top panel shows the data points from each ROI. OB of control group (blue), OB of α-synuclein group (yellow), VC of control group (green and red), VC of α-synuclein group (purple and brown), SC of control group (pink and grey), SC of α-synuclein group (chartreuse and turquois). The ROI as designated as contra- (contralateral) or ipsi- (ipsilateral) relative to the injection and expression of α-synuclein. The lower panel shows the difference of response when the response of the olfactory bulb in the control group is subtracted from each ROI. BOLD responses from both the VC and SC differs significantly from the olfactory bulb. Asterisks refer to difference from control of *p*>0.05=not significant (ns), *p<0.05, **p<0.01, and ***p<0.001.

**Figure 4.**
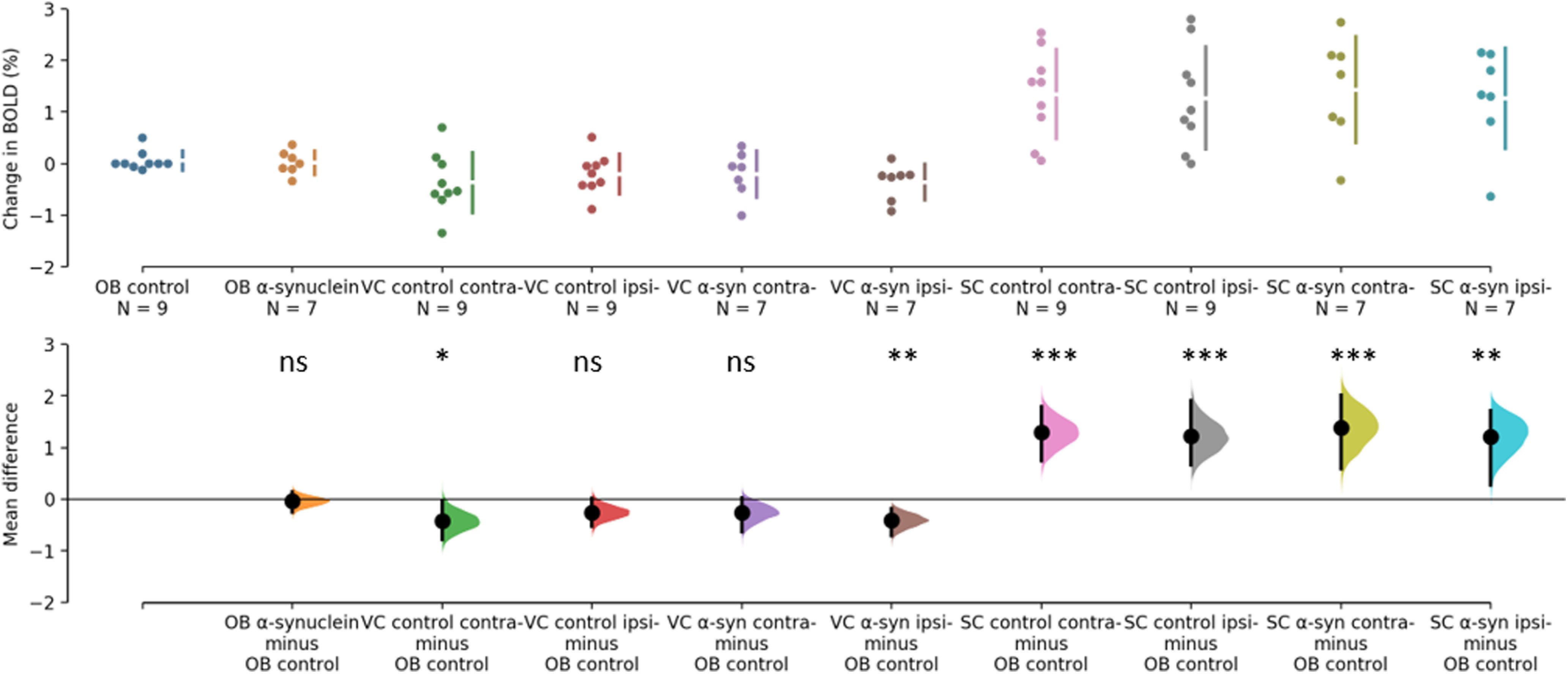
Estimation statistics of percent change in BOLD during 14 Hz. The top panel shows the data points from each ROI. OB of control group (blue), OB of α-synuclein group (yellow), VC of control group (green and red), VC of α-synuclein group (purple and brown), SC of control group (pink and grey), SC of α-synuclein group (chartreuse and turquois). The lower panel shows the difference of response when the response of the olfactory bulb in the control group is subtracted from each ROI. Responses from the SC differ significantly from OB. As does the contralateral VC from control group along with the response from the ipsilateral to the injection in the α-synuclein group. Asterisks refer to difference from control of *p*>0.05=not significant (ns), *p<0.05, **p<0.01, and ***p<0.001.

**Figure 5.**
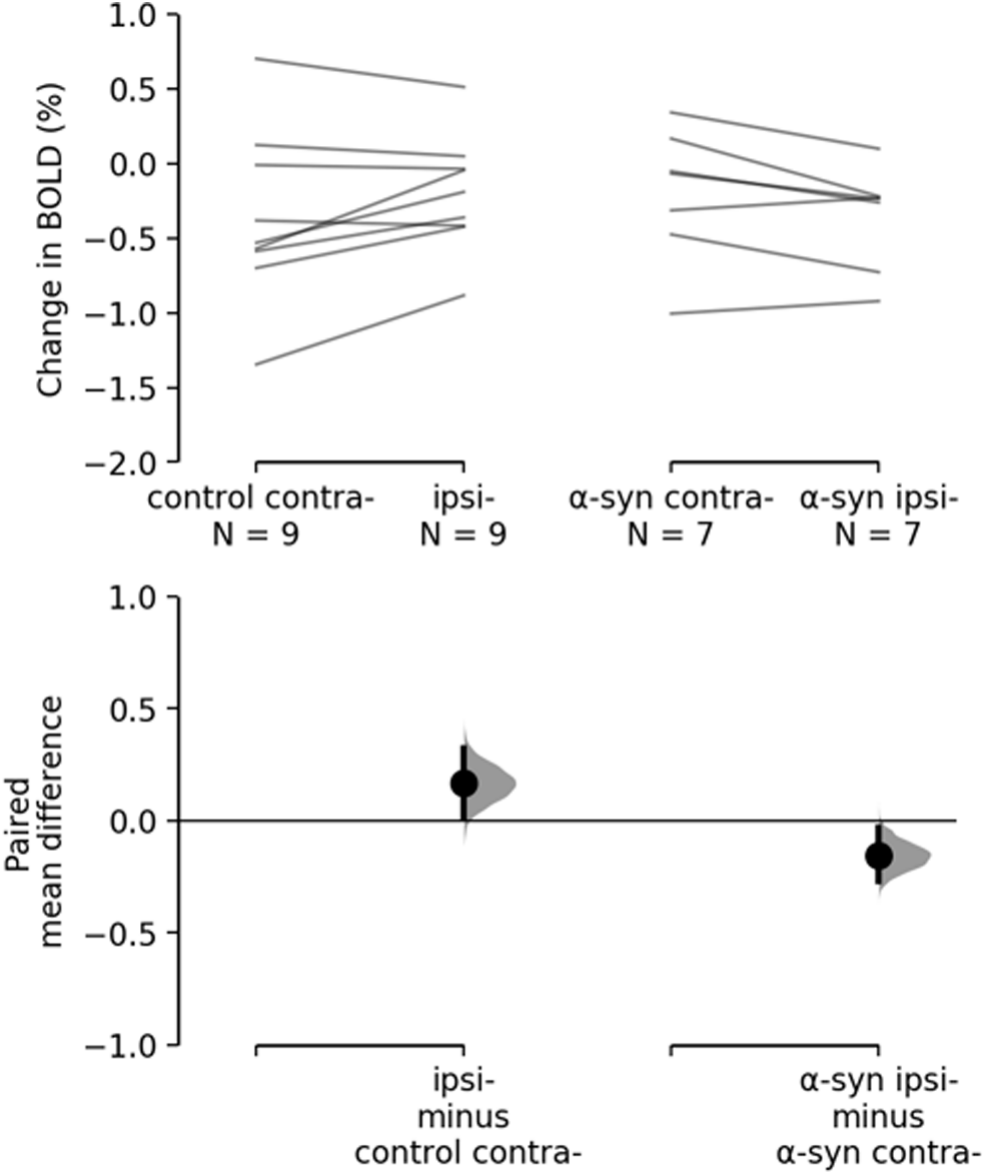
Comparison of differences within animals as paired. Right and left hemisphere from the control group, are shown on the left, and for the α-synuclein group on the right. The lower panel shows the estimated mean difference between hemispheres within the groups. The statistics does not reveal any asymmetry induced by expression of human α-synuclein.

The BOLD changes from each of the ROIs are visualized as points in the top panels of Figures 3, 4. Figure 3 shows the responses to light flashing at 1 Hz. Estimation statistics comparing each ROI to the OB revealed a significant response in both the VC and SC to the light flashing at 1 Hz (Fig. 3). Table 1 lists the full results with effects sizes and CIs. Figure 4 shows the responses to 14-Hz flashing light. There was a statistically significant response to the light stimulation in the SC. Furthermore, a significant difference was shown for VC ipsilateral to the injection of α-synuclein in the 14-Hz condition, with a magnitude of −0.414 (CI[−0.714,−0.182], *p* = 0.0012). However, there was also a difference in the control with an effect size of −0.424 (CI[−0.785,−0.0208], *p* = 0.0496; Fig. 4). Taken together, this suggests that the effect was not induced by the expression of α-synuclein. Regardless, this potential asymmetry was explored by comparing the VC within animals with a paired, permutation *t* test (Fig. 5). The control group showed an insignificant difference between hemispheres measured in the VC effect size of 0.167 (CI[0.0163,0.324], *p* = 0.0932). Similarly, there was no detectable difference between hemispheres in the α-synuclein group effect size −0.156 (CI[−0.268,−0.0296], *p* = 0.064). In conclusion, the expression of the α-synuclein did not induce detectable changes in the BOLD response within animals.

## Discussion

This study examined whether expression of α-synuclein in the SNc is associated with abnormal visually evoked brain activation. Using estimation statistics, we found robust BOLD-fMRI responses to the visual stimulus in the SC in both control rats and α-synuclein rats. The BOLD response in the VC turned out to be dependent on the presentation rate of the stimulus. A fully developed expression of α-synuclein in the SNc, in the optic layer of SC, and in the striatum did not induce any detectable changes in BOLD-fMRI during visual stimulation. This indicates that the level of neuronal activity measured during visual stimulation was not altered by expression of α-synuclein. Alternatively, the scan protocol used for BOLD-fMRI at 7T was not sensitive enough to detect any subtle differences between groups. However, the BOLD responses in rat visual system reproduced the frequency-dependence of the rodent visual cortex that was previously reported (Van Camp et al., 2006). For VC, the visually evoked BOLD responses tended to decrease at stimulation frequencies above 10 Hz, similar to the data shown in another study (Bailey et al., 2013). The BOLD signal in the SC did not show this frequency-response behavior.

The AAV-model was validated both behaviorally and with immunohistochemistry. Previously, asymmetry in the cylinder test have been shown to be specific to α-synuclein expression and is not an effect of induced protein expression with, e.g., green fluorescent protein (Andersen et al., 2018). In the present study, it is shown that there is indeed asymmetry in the use of forepaws as measured in the cylinder test.

After the scan sessions the brains of each of the animals were stained for human wild-type α-synuclein to validate that the model was developed. Both the cylinder test and the immunohistochemistry confirmed a successful development of human α-synuclein expression in the SNc and connected areas such as the striatum and the SC of the α-synuclein animals. Furthermore, it showed that human α-synuclein was present in the SC ipsilateral to the injection, however, this did not cause a detectable asymmetry in the BOLD-response of the SC on visual stimulation. Previously, the effect of human α-synuclein expression in rats was measured using *in vivo* electrophysiology. Here, subtle changes in the latency of VEPs in the SC were shown (Østergaard et al., 2020). The temporal resolution of fMRI might not be suitable to detect subtle increases in latency. This may explain the lack of difference between the responses in the two hemispheres in the α-synuclein animals. There is no direct correlation between event-related potentials and BOLD-fMRI but the γ-band power (Huettel et al., 2004) and event-related spectral perturbations (Engell et al., 2012) have been shown to correlate with the BOLD response. The results in this study support this discrepancy between the techniques.

Interestingly, there was a significant difference in the VC compared with the OB in the 14-Hz stimulation. A decreased BOLD response was observed in the VC ipsilateral to the injection in the α-synuclein group and in the VC contralateral to the injection in the control group compared with the OB. However, this was not because of asymmetry between the hemispheres in any of the groups, suggesting that the observed effect was not caused by the expression of α-synuclein.

In this study, the rodent eye is not considered to be implicated. Also, the study by Østergaard et al. (2020) did not show any statistically significant changes in the visual cortex after injection of AAV carrying human α-synuclein. If the rodent eye was functionally impacted, then both the SC and the VC would also have been affected. The AAV virus was injected in the SNc and the virus particles tend to spread in the area proximal to the injection site and via direct neuronal projections (Albert et al., 2019). Furthermore, the SNc is not a retinorecipient region of the rat visual system (Sefton et al., 2014).

The AAV α-synuclein rat model of PD is not known to cause large anatomic changes in the brain, however, it does show a loss of dopaminergic cells (Decressac et al., 2012). A study by Kuebler and colleagues suggests that the change in dopamine can be measured by fMRI/PET using an amphetamine challenge (Kuebler et al., 2017). Generally, the functional consequences of expressing human α-synuclein *in vivo* have been shown with other MR-based methods than BOLD-fMRI. A study using diffusion kurtosis imaging imaged α-synuclein in transgenic mice (Khairnar et al., 2017). This technique evaluates structural changes instead of the changes in oxygen metabolism. Another study used MR spectroscopy to show that bilaterally overexpressing α-synuclein in the striatum caused changes in energy metabolism in rats (Cuellar-Baena et al., 2016). This technique is sensitive to changes in amounts of metabolites where BOLD detects changes in the oxygen metabolism of larger populations of neurons.

In rodent models of Alzheimer’s disease, changes have been reported in functional connectivity patterns at early stages of protein aggregation. This has been observed using resting state fMRI (Grandjean et al., 2014). It can be speculated that this could be caused by protein aggregation and that the specific composition of proteins may be less important. Thus, evaluation of the AAV-model using functional connectivity should be investigated further.

In human patients, Zhao and colleagues have described changes in the BOLD signal of visual areas concerned with the perception of movement (Zhao et al., 2014). Further studies would be needed to study whether motion perception is also affected in the AAV-model. Here, we studied low-level visual processing but higher order functions such as motion perception may be more sensitive to the presence of α-synuclein. Generally, task-related BOLD-fMRI is rarely used to study PD in animals and humans. Often resting state fMRI and diffusion MRI are applied (Lehericy et al., 2017; Tessitore et al., 2019).

The necessity of anesthesia is a major challenge for fMRI studies in rodents. Anesthesia generally works by altering the neuronal activity (Masamoto and Kanno, 2012). Consequently, it will also affect the BOLD response. Unlike urethane and α-chloralose, a mix of isoflurane and dexmedetomidine may be used in longitudinal studies. The anesthesia paradigm used in the present study showed detectable and robust activation of the SC 3 h after the induction of anesthesia. As the functional changes in the SC are believed to be subtle, it cannot be ruled out that the effect of the anesthesia may mask potential functional differences.

In summary, this study shows that the fully developed expression of α-synuclein in the SNc along with the optic layer of SC and the striatum, did not induce any asymmetry detectable using BOLD-fMRI during visual stimulation.

## Synthesis

Reviewing Editor: Kirill Martemyanov, The Scripps Research Institute Decisions are customarily a result of the Reviewing Editor and the peer reviewers coming together and discussing their recommendations until a consensus is reached. When revisions are invited, a fact-based synthesis statement explaining their decision and outlining what is needed to prepare a revision will be listed below. The following reviewer(s) agreed to reveal their identity: Tristan Chaplin, Hossein Aleyasin, Kiyoshi Ando.

Minor:

Line 53: The statement “... the superior colliculus (SC) which is the primary visual nucleus of rodents” needs a clarification of what is meant by nucleus and a reference for its primacy (i.e. why not V1, why does it need to be a nucleus etc). e.g. “the superior colliculus (SC) which is the primary visual processing brain region of rodents [citation]”.

However, one of the issues that I raised about the possibility of α-synuclein aggregation in retina still remains unresolved. This is important since α-synuclein aggregation and damage to TH+ amacrine cells of the retina have been reported in cases of clinical PD and animal models of Parkinson's disease (reviewed by Indrieri et al., PMID: 33192254). I appreciate and understand that it is “unlikely” that it is the case in this study, but it is necessary to directly discuss and address the issue in the paper. The best approach still would be to histologically show lack of retinal injury and absence of α-synuclein aggregates in retina, which is unfortunately missing from the Østergaard et al 2020 study too. The next possible approach is to include the indirect implication of Østergaard et al 2020 results preferably in the discussion section, by citing the lack of abnormal response in VC.

## References

[B1] Albert K, Voutilainen MH, Domanskyi A, Piepponen TP, Ahola S, Tuominen RK, Richie C, Harvey BK, Airavaara M (2019) Downregulation of tyrosine hydroxylase phenotype after AAV injection above substantia nigra: caution in experimental models of Parkinson’s disease. J Neurosci Res 97:346–361. 30548446 10.1002/jnr.24363 PMC11863348

[B2] Andersen MA, Christensen KV, Badolo L, Smith GP, Jeggo R, Jensen PH, Andersen KJ, Sotty F (2018) Parkinson’s disease-like burst firing activity in subthalamic nucleus induced by AAV-α-synuclein is normalized by LRRK2 modulation. Neurobiol Dis 116:13–27. 10.1016/j.nbd.2018.04.011 29680709

[B3] Bailey CJ, Sanganahalli BG, Herman P, Blumenfeld H, Gjedde A, Hyder F (2013) Analysis of time and space invariance of BOLD responses in the rat visual system. Cereb Cortex 23:210–222. 10.1093/cercor/bhs008 22298731 PMC3513959

[B4] Bodis-Wollner I, Kozlowski PB, Glazman S, Miri S (2014) a-Synuclein in the inner retina in Parkinson disease. Ann Neurol 75:964–966. 10.1002/ana.24182 24816946

[B5] Cox RW (1996) AFNI: software for analysis and visualization of functional magnetic resonance neuroimages. Comput Biomed Res 29:162–173. 10.1006/cbmr.1996.0014 8812068

[B6] Cuellar-Baena S, Landeck N, Sonnay S, Buck K, Mlynarik V, In ’T, Zandt R, Kirik D (2016) Assessment of brain metabolite correlates of adeno-associated virus-mediated over-expression of human alpha-synuclein in cortical neurons by in vivo (1)H-MR spectroscopy at 9.4 T. J Neurochem 137:806–819. 10.1111/jnc.13547 26811128

[B7] Davidsdottir S, Cronin-Golomb A, Lee A (2005) Visual and spatial symptoms in Parkinson’s disease. Vision Res 45:1285–1296. 10.1016/j.visres.2004.11.006 15733961

[B8] Decressac M, Mattsson B, Lundblad M, Weikop P, Björklund A (2012) Progressive neurodegenerative and behavioural changes induced by AAV-mediated overexpression of α-synuclein in midbrain dopamine neurons. Neurobiol Dis 45:939–953. 10.1016/j.nbd.2011.12.013 22182688

[B9] Ekker MS, Janssen S, Seppi K, Poewe W, de Vries NM, Theelen T, Nonnekes J, Bloem BR (2017) Ocular and visual disorders in Parkinson’s disease: common but frequently overlooked. Parkinsonism Relat Disord 40:1–10. 10.1016/j.parkreldis.2017.02.014 28284903

[B10] Engell AD, Huettel S, McCarthy G (2012) The fMRI BOLD signal tracks electrophysiological spectral perturbations, not event-related potentials. Neuroimage 59:2600–2606. 10.1016/j.neuroimage.2011.08.079 21925278 PMC3277784

[B11] Grandjean J, Schroeter A, He P, Tanadini M, Krstic D, Keist R, Konietzko U, Klohs J, Nitsch RM, Rudin M (2014) Early alterations in functional connectivity and white matter structure in a transgenic mouse model of cerebral amyloidosis. J Neurosci 34:13780–13789. 10.1523/JNEUROSCI.4762-13.2014 25297104 PMC6608375

[B12] Ho J, Tumkaya T, Aryal S, Choi H, Claridge-Chang A (2019) Moving beyond P values: data analysis with estimation graphics. Nat Methods 16:565–566. 10.1038/s41592-019-0470-3 31217592

[B13] Huettel SA, McKeown MJ, Song AW, Hart S, Spencer DD, Allison T, McCarthy G (2004) Linking hemodynamic and electrophysiological measures of brain activity: evidence from functional MRI and intracranial field potentials. Cereb Cortex 14:165–173. 10.1093/cercor/bhg115 14704213

[B14] Khairnar A, Ruda-Kucerova J, Szabó N, Drazanova E, Arab A, Hutter-Paier B, Neddens J, Latta P, Starcuk Z, Rektorova I (2017) Early and progressive microstructural brain changes in mice overexpressing human α-Synuclein detected by diffusion kurtosis imaging. Brain Behav Immun 61:197–208. 10.1016/j.bbi.2016.11.027 27923670

[B15] Kjonigsen LJ, Lillehaug S, Bjaalie JG, Witter MP, Leergaard TB (2015) Waxholm Space atlas of the rat brain hippocampal region: three-dimensional delineations based on magnetic resonance and diffusion tensor imaging. Neuroimage 108:441–449. 10.1016/j.neuroimage.2014.12.080 25585022

[B16] Klein C, Westenberger A (2012) Genetics of Parkinson’s disease. Cold Spring Habor Perspect Med 2:1–15. 10.1101/cshperspect.a008888 PMC3253033 22315721

[B17] Kuebler L, Herfert K, Landeck N, Maurer A, Amend M, Thielcke A, Buss S, Marciano S, Stumm R, Wehrl HF, Kirik D, Pichler BJ (2017) Quantification of molecular and functional changes in a rat model of Parkinson’s disease using a simultaneaous PET/fMRI protocol. Available at https://www.abstractsonline.com/pp8/index.html#!/4376/presentation/33702.

[B18] Lehéricy S, Bardinet E, Poupon C, Vidailhet M, François C (2014) 7 tesla magnetic resonance imaging: a closer look at substantia nigra anatomy in Parkinson’s disease. Mov Disord 29:1574–1581. 10.1002/mds.26043 25308960

[B19] Lehericy S, Vaillancourt DE, Seppi K, Monchi O, Rektorova I, Antonini A, McKeown MJ, Masellis M, Berg D, Rowe JB, Lewis SJG, Williams-Gray CH, Tessitore A, Siebner HR (2017) The role of high-field magnetic resonance imaging in parkinsonian disorders: pushing the boundaries forward. Mov Disord 32:510–525. 10.1002/mds.26968 28370449

[B20] Masamoto K, Kanno I (2012) Anesthesia and the quantitative evaluation of neurovascular coupling. J Cereb Blood Flow Metab 32:1233–1247. 10.1038/jcbfm.2012.50 22510601 PMC3390804

[B21] Niethammer M, Feigin A, Eidelberg D (2012) Functional neuroimaging in Parkinson’s disease. Cold Spring Harb Perspect Med 2:1–21. 10.1101/cshperspect.a009274 22553499 PMC3331691

[B22] Noyce AJ, Lees AJ, Schrag A-E (2016) The prediagnostic phase of Parkinson’s disease. J Neurol Neurosurg Psychiatry 87:871–878. 10.1136/jnnp-2015-311890 26848171 PMC4975823

[B23] Østergaard FG, Himmelberg MM, Laursen B, Siebner HR, Wade AR, Christensen KV (2020) Classification of α-synuclein-induced changes in the AAV α-synuclein rat model of Parkinson’s disease using electrophysiological measurements of visual processing. Sci Rep 10:1–14. 32681050 10.1038/s41598-020-68808-3 PMC7368019

[B24] Papp EA, Leergaard TB, Calabrese E, Johnson GA, Bjaalie JG (2014) Waxholm Space atlas of the Sprague Dawley rat brain. Neuroimage 97:374–386. 10.1016/j.neuroimage.2014.04.001 24726336 PMC4160085

[B25] Pawela CP, Biswal BB, Hudetz AG, Schulte ML, Li R, Jones SR, Cho YR, Matloub HS, Hyde JS (2009) A protocol for use of medetomidine anesthesia in rats for extended studies using task-induced BOLD contrast and resting- state functional connectivity. Neuroimage 46:1137–1147. 10.1016/j.neuroimage.2009.03.004 19285560 PMC2693293

[B26] Paxinos G, Watson C (1998) The rat brain: in stereotaxic coordinates, Ed 4. San Diego; London: Academic Press.

[B27] Pyatigorskaya N, Gallea C, Garcia-Lorenzo D, Vidailhet M, Lehericy S (2014) A review of the use of magnetic resonance imaging in Parkinson’s disease. Ther Adv Neurol Disord 7:206–220. 10.1177/1756285613511507 25002908 PMC4082302

[B28] Ridder A, Müller MLTM, Kotagal V, Frey KA, Albin RL, Bohnen NI (2017) Impaired contrast sensitivity is associated with more severe cognitive impairment in Parkinson disease. Parkinsonism Relat Disord 34:15–19. 10.1016/j.parkreldis.2016.10.006 27742131 PMC5222688

[B29] Satue M, Rodrigo MJ, Obis J, Vilades E, Gracia H, Otin S, Fuertes MI, Alarcia R, Crespo JA, Polo V, Larrosa JM, Pablo LE, Garcia-Martin E (2017) Evaluation of progressive visual dysfunction and retinal degeneration in patients with Parkinson’s disease. Invest Ophthalmol Vis Sci 58:1151–1157. 10.1167/iovs.16-20460 28208185

[B30] Sefton AJ, Dreher B, Harvey AR, Martin PR (2014) Visual system, the rat nervous system, Ed 4. Cambridge, Massachusetts: Academic Press.

[B31] Sergejeva M, Papp EA, Bakker R, Gaudnek MA, Okamura-Oho Y, Boline J, Bjaalie JG, Andreas H (2015) Anatomical landmarks for registration of experimental image data to volumetric rodent brain atlasing templates. J Neurosci Methods 240:161–169. 10.1016/j.jneumeth.2014.11.005 25445058

[B32] Tessitore A, Cirillo M, De Micco R (2019) Functional connectivity signatures of Parkinson’s disease. J Parkinsons Dis 9:637–652. 10.3233/JPD-191592 31450512 PMC6839494

[B33] Thomas B, Beal MF (2007) Parkinson’s disease. Hum Mol Genet 16:R183–R194. 10.1093/hmg/ddm159 17911161

[B34] Van Camp N, Verhoye M, De Zeeuw CI, Van der Linden A (2006) Light stimulus frequency dependence of activity in the rat visual system as studied with high-resolution BOLD fMRI. J Neurophysiol 95:3164–3170. 10.1152/jn.00400.2005 16394078

[B35] Weil RS, Schrag AE, Warren JD, Crutch SJ, Lees AJ, Morris HR (2016) Visual dysfunction in Parkinson’s disease. Brain 139:2827–2843. 10.1093/brain/aww175 27412389 PMC5091042

[B36] Wirdefeldt K, Adami H, Cole P, Trichopoulos D, Mandel J (2011) Epidemiology and etiology of Parkinson’s disease: a review of the evidence. Eur J Epidemiol 26:S1–S58. 10.1007/s10654-011-9581-6 21626386

[B37] Woolrich MW, Jbabdi S, Patenaude B, Chappell M, Makni S, Behrens T, Beckmann C, Jenkinson M, Smith SM (2009) Bayesian analysis of neuroimaging data in FSL. Neuroimage 45:S173–S186. 10.1016/j.neuroimage.2008.10.055 19059349

[B38] Zhao Y, Zheng X, Wang Q, Xu J, Xu X, Zhang M (2014) Altered activation in visual cortex: unusual functional magnetic resonance imaging finding in early Parkinson’s disease. J Int Med Res 42:503–515. 10.1177/0300060513507647 24595154

